# Effects of Environmental Pollutants on Tryptophan Metabolism

**DOI:** 10.3390/toxics13040311

**Published:** 2025-04-17

**Authors:** Hongyang Hu, Xiaoxun Lu, Miaoliang Wu, Zhi Bai, Xiaoshan Liu

**Affiliations:** School of Public Health, The First Dongguan Affiliated Hospital, Guangdong Medical University, Dongguan 523808, China; 18589259270@163.com (H.H.); 17865587308@163.com (X.L.); wml22821677441@163.com (M.W.); weidayou100@163.com (Z.B.)

**Keywords:** tryptophan, kynurenine pathway, 5-HT pathway

## Abstract

Tryptophan (Trp) is an important essential amino acid that plays a variety of physiological functions in the human body, including being a precursor of neurotransmitter and participating in immune regulation. Currently, more and more studies show that some pollutants in the environment can affect the metabolism of Trp and consequently affect human health. The present paper offers a comprehensive overview of prior research investigating the impact of environmental pollutants, including inorganic and organic contaminants, microplastics, and nanoplastics on the nervous system, immune system, digestive system, and maternal–fetal pregnancy, revealing their detrimental effects on Trp metabolism and human well-being.

## 1. Introduction

With the accelerated development of industrialization and urbanization, environmental pollution has become a major challenge facing the world, and its impact on human health has drawn increasing attention. Trp, an important essential amino acid, plays various physiological functions in the human body, including being a precursor of neurotransmitter and playing a key role in immune regulation. Trp metabolism, especially serotonin, which is the most relevant neurotransmitter, plays an essential role in various physiological systems, including the nervous system, immune regulation, gut homeostasis, and maternal–fetal health. Disruptions in Trp metabolism are linked to depression, neurodegenerative diseases, inflammatory bowel diseases, and pregnancy complications, all of which impose a significant burden on public health globally. In recent years, an increasing number of studies have shown that environmental pollutants affect human health by influencing Trp metabolism.

This manuscript aims to provide a comprehensive review of current research, focusing on common pollutant types, their mode of action, and the latest findings regarding their potential health impacts. By shedding light on these issues, this review seeks to contribute to the development of evidence-based strategies for pollution control and public health interventions.

## 2. Material and Methods

The articles search strategy is as follows. First, identifying the research question and Keywords. The research question is the impact of environmental pollutants on Trp metabolism and its potential hazards to human health. Based on this, the following keywords were identified. Environmental pollutants (including heavy metals, organic pollutants, microplastics, and nanoplastics), trp metabolism (including the kynurenine (KYN) pathway and 5-HT pathway), and health impacts (including neurological disorders, immune system, digestive system, and maternal–fetal health). PubMed and Web of Science were used to select articles. Logical operators (AND/OR/NOT) were used to combine keywords into search strategies to improve both precision and comprehensiveness. Then, the literature was screened according to following criteria: (1) articles directly examining the impact of environmental pollutants on Trp metabolism, involving Trp metabolic pathways (KYN and 5-HT pathways) and their roles in various physiological systems (nervous, immune, digestive systems, and maternal health); (2) articles including original research, reviews, and meta-analyses, with a preference for high-quality experimental studies and systematic reviews from the past 10 years (classical and foundational research is included regardless of publication date); (3) articles demonstrating scientific rigor and reliability, with clearly defined methods and results. Preference is given to articles published in high-impact journals, while articles unrelated to the effects of environmental pollutants on Trp metabolism or those addressing Trp metabolism without involving environmental pollutants as well as duplicate publications or articles with highly similar content are excluded. The literature search strategy is shown in Table 1.

## 3. Tryptophan

### 3.1. Source of Trp

**Figure 1:**
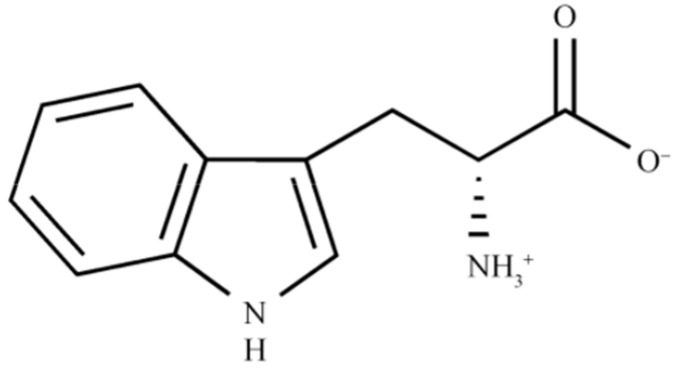
The structural formula of Trp.

Trp was first isolated from casein by Hokinst in 1902, with a molecular formula of C_11_H_12_N_2_O_2_ and a molecular mass of 204.2. The structural formula of Trp is shown in Figure 1. Trp has three isomers: L, D, and racemic DL [1]. The L-type is a flake crystal that is odorless. The D-type and spiral DL type are white crystals, with the D-type having a distinctive taste. The L isomer is virtually the only one that occurs in nature. In summary, Trp is one of the eight essential amino acids in the human body and is the only amino acid that binds to plasma albumin and exists in a balanced state between the albumin-bound and free forms in the peripheral circulation. As an essential amino acid, Trp is typically obtained through daily intake of meat, milk, eggs, and seed-based foods such as soy, sesame, and sunflower seeds [2]. It plays a key role in protein biosynthesis and serves as a precursor for the synthesis of a variety of important bioactive compounds.

### 3.2. The Pathway of Trp Metabolism

**Figure 2:**
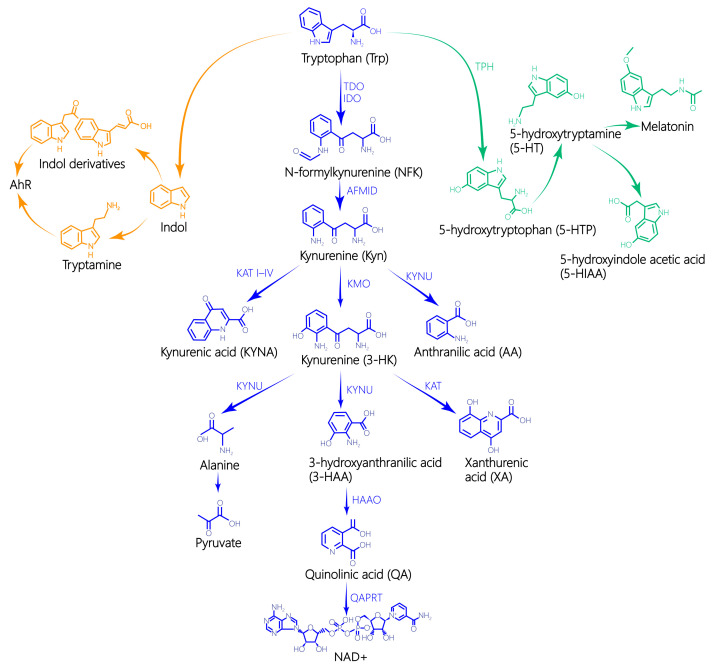
L-tryptophan metabolite pathway. The full names of the abbreviations in the Figure 2 are as follows: TPH—Trp hydroxylase; IDO—indoleamine-2,3-dioxygenase; TDO—Trp-2,3-dioxygenase; AFMID—arylformamidase; KAT—KYN aminotransferases; KYNU—kynureninase; KMO—kynurenine-3-monooxygenase; HAAO—3-hydroxyanthranilate-3,4-dioxygenase; QAPRT—QA phosphoribosyltransferase; AhR—aryl hydrocarbon receptor.

There are three metabolic pathways of Trp: the KYN pathway, the 5-hydroxytryptamine (5-HT) pathway, also known as serotonin pathway, and the indoleacetic acid pathway [3,4] (Figure 2). More than 95% of the free Trp in the body is converted into KYN by indoleamine 2,3-dioxygenase (IDO) and tryptophane-2,3-dioxygenase (TDO), which is then catalyzed by various catalytic enzymes to produce 3-hydroxykynurenine (3-HK), 3-hydroxyanthranilic acid (3-HAA), and quinolinic acid (QA). In turn, nicotinamide adenine dinucleotide (NAD+) is subsequently produced, which can activate N-methyl-D-aspartic acid (NMDA) receptors, oxidative stress, and neuroinflammation, leading to glutamate-driven excitatory toxicity. In addition, KYN can be metabolized into kynurenic acid (KYNA) and anthranilic acid (AA), both of which have immune regulatory and neuroprotective effects [5]. Recent studies have demonstrated that after Daphnia magna were exposed to 0.01, 0.05, and 0.09 mg/L cadmium for 24 h and 48 h, the content of 5-HIAA was significantly lower than the control group, and there was a concentration-effect relationship [6].

The remaining 5% of Trp can be metabolized by TPH to produce 5-HT, which is a crucial neurotransmitter in the central nervous system and plays a regulatory role in several physiological systems [7]. Subsequently, 5-HT can be oxidized by monoamine oxidase (MAO) to produce 5-hydroxyindoleacetic acid (5-HIAA), which is excreted in the urine [8]. Trp metabolism mainly affects the nervous system, immune system, digestive system, maternal and fetal pregnancy, and so on. Moreover, the gut microbiota converts trace amounts of Trp into indole and its derivatives, such as indole-acrylic acid, indole-3-acetic acid (IAA), indole-3-propionic acid (IPA), indole-3-acetaldehyde (3-IAld), and tryptamine [9,10]. Thus, Trp metabolism influences various physiological processes such as neuron function, immune response, intestinal homeostasis, and maternal–fetal tolerance. The significance of Trp in the human body should not be underestimated.

## 4. The Effect of Trp Metabolism in Various Physiological Systems

### 4.1. Nervous System

Previous studies have shown that once Trp crosses the blood–brain barrier and enters the extracellular or cerebrospinal fluid (CSF) of the central nervous system, it can be readily taken up by the cells of the central nervous system. In addition to serotonergic neurons, astrocytes, microglia, dendritic cells (DCs), and mast cells can use Trp as a precursor to synthesize neuroactive metabolites. Mast cells use Trp to synthesize 5-HT [11]. This 5-HT may contribute to neuroimmune interactions in the brain, including regulation of blood–brain barrier permeability [12]. Furthermore, pineal cells synthesize 5-HT from Trp, which acts as a precursor to the synthesis of melatonin. Consequently, melatonin is subsequently released from the cell and acts as a neurohormone affecting the function of the central nervous system [13]. As a neurotrophic factor, 5-HT plays an important role in the process of brain development, and as a major regulator of the behavior of vertebrates and invertebrates, it is related to various physiological functions of the central nervous system. It is critical for in mood, sleep, cognition, memory, appetite, executive function, social behavior, and sensory gating. Thus, 5-HT is a major therapeutic target for patients with mental illness [14,15].

In the meantime, astrocytes, microglia, and DCs metabolize Trp through the KYN pathway [16]. The products of Trp metabolism through the KYN pathway include the neuroactive free radical generators 3-HK and 3-HAA [2,17], excitotoxic NMDA receptor agonists and free radical generators QA [18], and the neuroprotective NMDA and α7 nicotinic acetylcholine receptor antagonist KYNA [19,20]. A previous study in urethane-anesthetized (1.5 g/kg, intraperitoneal injection) male Wistar rats demonstrated that quinolinic acid (QA) selectively activates NMDA-sensitive glutamate receptor subtypes. This activation not only excites neurons but also induces neuronal damage through receptor overstimulation [21]. 3-HK (and its metabolite 3-HAA) is less toxic than QA. The resulting neuronal damage is mediated by free radicals rather than through glutamate receptors [22], and 3-HK can induce neuronal cell death, especially in cortical and striatal neurons [23]. KYNA acts as an antagonist of the NMDA receptor and has been found to be particularly effective as an antagonist of the allosteric site of the NMDA receptor [24]. Based on this property, compounds with potential therapeutic value in stroke, epilepsy, and neurodegenerative diseases have been developed clinically [2,18,25]. There is evidence that melatonin synthesis in the pineal gland changes significantly in a circadian manner with high levels of synthesis and secretion during the dark phase of the circadian cycle and is considered to be an important neuroendocrine signal that transmits circadian and seasonal information to multiple organ systems, including the brain, which regulates many physiological and behavioral responses, resulting in sexual maturation and reproductive behavior, thermoregulation, sleep patterns, metabolism, hematopoietic, immune response, and reduction of oxidative stress [26,27]. It can be seen that Trp metabolism is essential for the normal functioning of the nervous system and the homeostatic environment.

### 4.2. Immune System

The role of Trp in the immune system mainly focuses on tumor immune tolerance and inflammatory response [28]. DCs are responsible for initiating immunity, maintaining autotolerance, and controlling overactivated inflammatory states, and IDO1 expression in this cell is induced by inflammatory stimuli, including type I and type II interferons, lipopolysaccharides (LPS), and extracellular and intracellular DNA [5]. Studies have shown that higher levels of IDO1 are associated with higher immunosuppressive activity of Treg cells in tumors [29], and IDO1-mediated Trp depletion in DCs can inhibit CD8+ T cell activation and transform naive CD4+ T cells into Treg cells through amino acid hunger sensor general control nonderepressible 2 (GCN2) kinase [30]. Additionally, IDO1-induced GCN2 activation is necessary for splenic macrophages to produce interleukin-10 (IL-10) and transforming growth factor-β (TGF-β) and induce peripheral tolerance to apoptotic cells [31]. Interferon-γ (IFN-γ) from donor CD4+ T cells and host inflammatory cytokines (including IL-1β and tumor necrosis factor-α (TNF-α)) induce IDO1 and aryl hydrocarbon receptor (AhR) expression, respectively, by activating nuclear factor kappa-light-chain-enhancer of activated B cells (NF-κB). This cell-intrinsic IDO1-AHR pathway inhibits STA1-mediated IL-6 expression and subsequently prevents CD4+ T cells from differentiating into pathogenic Th17 cells [32,33]. Ravishankar et al. also revealed that IDO1 activation plays a central role in maintaining peripheral self-tolerance to apoptotic cells, which will lead to systemic lupus erythematosus (SLE) [34] when this process is abnormal.

For the 5-HT pathway, peripheral TPH1 converts Trp to 5-hydroxytryptophan (5-HTP), which is a precursor to 5-HT and melatonin. IL-2 induces TPH1 expression in CD8+ T cells by continuously activating signal transducer and activator of transcription 5 (STAT5) in the tumor, while 5-HTP in turn activates AhR in CD8+ T cells, which enables them to acquire a depletion phenotype, resulting in synergistic upregulation of inhibitory receptors. This study suggested that the tumor microenvironment establishes an immune escape mechanism. This mechanism is related to negative feedback regulation initiated by powerful immune stimulants. Other Trp metabolites, including 5-HT, N-acyl 5-HT, and melatonin, have anti-inflammatory and immunomodulatory properties [35].

Furthermore, 5-HT receptors are important for tumor angiogenesis and serve as pro-mitosis and anti-apoptotic signals, thereby promoting tumor growth [36]. Previous studies generally believed that 5-HT in platelets can promote the aggregation of neutrophils to the site of acute inflammation, but in fact, 5-HT itself has no chemotactic ability for neutrophils [37]. In a recent study, DeGiovani et al. demonstrated that the 5-HT metabolite 5-HIAA is responsible for neutrophil chemotaxis [38]. 5-HIAA is released by platelets and mast cells at the site of infection, binds to G protein-coupled receptor 35 (GPR35) and causes its expression to be upregulated. GPR35 signaling promotes neutrophils to pass through the platelet-coated endothelial cell layer, and these neutrophils are further attracted to the site of infection in response to 5-HIAA released by the tissue mast cells. 5-HT regulates the immune response through its expression of 5-HT receptors on a variety of immune and non-immune cells [39].

### 4.3. Digestive System

The 5-HT pathway is one of the core signaling pathways in the gut, which plays an important role in regulating intestinal permeability and mucosal inflammation. Studies have found that gastrointestinal selective TPH inhibitors can manage several gastrointestinal diseases [40], inhibiting mucosal 5-HT production alleviates inflammation [41], and microbial short-chain fatty acids (SCFAs) can promote the TPH1 transcription of enterochromaffin cells and the production of colon 5-HT and stimulate colon transport [42]. Recent studies have explored the role of melatonin in the gut, especially its role in intestinal permeability, which can mitigate the increase in intestinal permeability and immune activation caused by *Escherichia coli* [43], and the protective effect of melatonin on induced intestinal permeability is at least partially mediated by α7nAChr [44]. There is growing evidence that Trp, and its endogenous host metabolites (KYN, 5-HT, and melatonin), have a profound effect on the interaction between the host immune system and the gut microbiota.

The gut microbiota and microbial metabolites are crucial for maintaining intestinal health, and the microbiota influences the development of the immune system, which in turn shapes the composition of the gut microbiota [45]. Trp and its endogenous metabolite participate in intestinal immune homeostasis, and microbiota can directly and indirectly regulate host endogenous Trp metabolism. Changes in Trp metabolism can affect microbial proliferation and microbial diversity, and insufficient dietary Trp will change intestinal microbial composition and damage intestinal immunity [46]. Trp has been shown to play an anti-inflammatory role in mammals, and itself, along with its regulatory pathways, are important regulators of inflammatory response [47]. Mice on a low-Trp diet are more likely to develop chemically induced intestinal inflammation [48]. Conversely, mice fed a diet with adequate amounts of Trp showed reduced inflammation and reduced severity of dextran sodium sulfate (DSS)-induced colitis [49]. In addition, when tissues are injured, intestinal monocytes overexpress IDO1, thereby regulating host immunomodulatory activity by promoting the production of KYN, maintaining mucosal amino acid nutrition, improving mucosal immune reactivity and changing intestinal microbial community metabolism, thus exerting anti-inflammatory and immunosuppressant effects on intestinal mucosa [50]. For Trp metabolites, KYN exhibits antibacterial activity that directly affects the proliferation of intestinal microbiota [51]. KYN acts as a direct ligand of AhR, and stimulates AhR and AhR-dependent gene expression in a concentration-dependent manner, while regulating intestinal homeostasis [52].

### 4.4. Pregnancy

In response to maternal protein synthesis and the growth and development of the fetus, the demand for Trp increases significantly during pregnancy. The placenta, as an important temporary organ, serves a variety of functions during pregnancy, including the acquisition of nutrients from maternal blood and the elimination of waste. It is also a secretory organ that can secrete a variety of hormones and cytokines and is also the main metabolic site of various amino acids at the mother–fetus interface [53]. Trp is also metabolized in the placenta through the 5-HT and KYN pathways, and the metabolites produced have different effects on the mother and fetus.

A landmark study by Boni et al., first identified the placenta as the source of 5-HT in the fetus, with 5-HT synthesis occurring at day 10.5 of the embryo (E10.5) in mice and at week 1 of gestation in humans [54]. Moreover, immunohistochemistry identified the expression of TPH1 in the syncytial trophoblasts of the mouse placenta, confirming that the placenta has the ability to synthesize 5-HT from Trp. Since the fetus itself cannot synthesize the required 5-HT through the brain in the early gestation period, 5-HT synthesized by the placenta during gestation is an important source for the normal development of the fetal nervous system [55]. Such dysfunction in its synthesis can negatively affect fetal brain development, such as causing autism spectrum disorder (ASD) [56]. Furthermore, 5-HT functions as a potent vasoconstrictor in the placenta and is important for maintaining placental blood flow during pregnancy [57].

**Figure 3:**
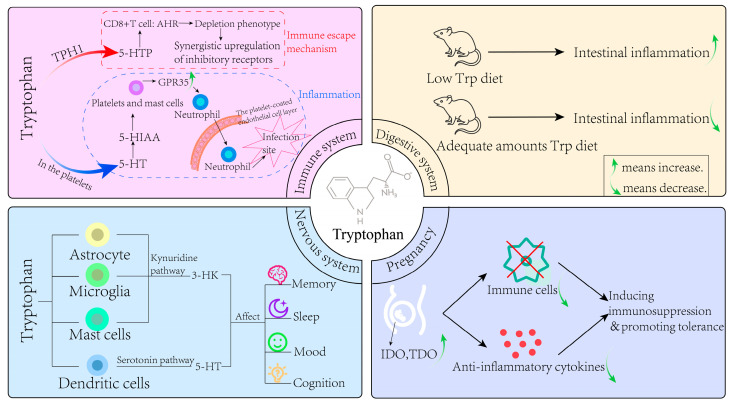
Role of Trp metabolism in human body.

As for the KYN pathway, it has been proven that metabolic enzymes related to this pathway are expressed in the placenta, and current studies mainly focus on its function in mediating placental immune tolerance [58]. The maternal–fetal tolerance mechanism is activated during pregnancy to allow embryo implantation and fetal development [59]. IDO and TDO are highly expressed in the placenta during pregnancy, resulting in increased levels of downstream kynuretic acid metabolites, promoting apoptosis of effector immune cells [60], and stimulating the production of anti-inflammatory cytokines, thus inducing immunosuppression and promoting tolerance [61]. There is ample evidence that KYN metabolites act through highly specific mechanisms involving their binding to AhR receptors [62], such as KYNA, a ligand of AhR, which helps establish an immunosuppressor environment and eliminate inflammation [63]. In addition to immune tolerance, this pathway also plays other roles, such as anti-infection, and umbilical vein endothelial cells can express IDO1, which results in antibacterial and anti-parasitic effects, helping to protect the fetus-placental unit from infection [64]. In addition, the catabolism of Trp by IDO1 in the chorionic membranes can induce vasodilation and help to send blood into the villous space, thus contributing to the maintenance of normal placental blood perfusion [65]. In Figure 3, we summarized the role of Trp metabolism in human health.

## 5. Effects of Environmental Pollutants on Trp Metabolism

**Figure 4:**
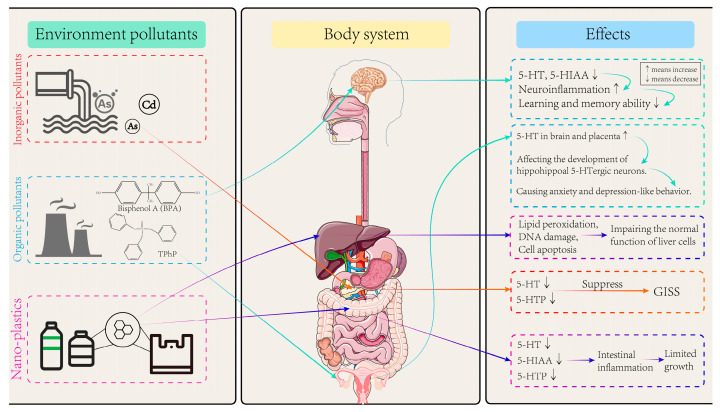
The impact of environmental pollutants on Trp metabolism.

Environmental pollutants are substances that enter the environment by man-made or non-human means and have a negative impact on ecosystems and human health. These pollutants can enter the environment through air, water, soil, etc., and their existence may lead to environmental degradation, destruction of ecological balance, and serious threat to human life [66]. Common environmental pollutants include heavy metals, nitrogen oxides, polycyclic aromatic hydrocarbons (PAHs), organic chlorine pesticides, bisphenol compounds (BPs), flame retardants, polychlorinated biphenyls (PCBs), microplastics, and so on (Figure 4). Environmental pollutants come from a wide range of sources, including industrial activities, agricultural production, urban development, transportation, and daily life, etc. Long-term exposure can lead to diseases, increase the risk of cancer, affect the function of the nervous system, and interfere with the endocrine system, posing a broad threat to human health [67].

### 5.1. Effects of Inorganic Pollutants on Trp Metabolism

Inorganic pollutants refer to those compounds or elements that do not contain carbon or only contain carbon in certain rare cases. Such pollutants include heavy metals (e.g., mercury (Hg), lead (Pb), cadmium (Cd), arsenic (As)), inorganic acids, bases, salts, and some inorganic gases (e.g., ammonia, sulfur dioxide, chlorine, ozone). These pollutants can enter water, soil, and the atmosphere through various pathways, such as industrial and agricultural activities, improper treatment of urban sewage and waste, and can have significant impacts on ecosystems and public health [68]. A mass spectrometry-based urine metabolomics study of people exposed to Cd through long-term consumption of locally sourced food over 15 years, with the minimum level of 3.08 ± 1.40 μg/L, has revealed that renal amino acid metabolism, particularly Trp metabolism, is primarily affected as urinary Cd concentrations increase [69]. Similarly, a case-control study found that the differential metabolites of plasma neurotransmitters detected in As-exposed populations were mainly concentrated in serotonergic, dopaminergic, and catecholaminergic pathways, and the results of mediation effect analysis indicated that Trp played a significant mediating role in the decrease in Mini-Mental State Examination (MMSE) scores caused by arsenic exposure [70].

An in vivo test, conducted on 80 male Wistar rats exposed to As at doses of 0.05, 0.25, 1.25, and 6.25 mg/L via oral administration for 30 days confirmed that As exposure can disrupt the balance of intestinal microflora and interfere with Trp metabolism in rats [71], inhibit the expression of TPH, reduce the level of 5-HT in the brain, and cause a significant reduction in the number of synaptic vesicles [72,73]. In the meanwhile, a study found that Zebrafish embryos exposed to mercury chloride (HgCl_2_) (0, 4, 40, 400 nM) via water exposure for up to 96 h showed reduced levels of three neurotransmitters, including tyrosine, dopamine, and Trp [74]. Additionally, another study conducted on male Fischer-344 rats exposed to 0.8 ppm ozone for 4 h found that ozone can reduce the level of Trp in serum, increase the KYN level, and decrease the expression of key neurotrophic factors in the hippocampus of rats, increasing the risk of anxiety and depression [75]. A recent study conducted on 7-week-old C57BL/6 male mice individually and jointly exposed to 25 mg/L Pb, 10 mg/L Hg, and 15 mg/L Cd for 28 days indicated that co-exposure to Pb and Cd significantly reduced KYNA levels in the brain, which are essential for neuroprotection, thus increasing the vulnerability to neurobehavioral disorders [76]. In vitro, a study demonstrated that 0.1–1 μM As exposure to MIN6-K8 mouse islet β cells 3 days significantly reduces the levels of 5-HT and its precursor 5-HTP, thereby triggering glucose-induced inhibition of insulin secretion (GIIS) [77]. These findings highlight the importance of policies limiting heavy metal contamination. Moreover, these exposures, especially when chronic and at low doses, can have cumulative, long-term effects on the Trp metabolic pathway.

### 5.2. Effects of Organic Pollutants on Trp Metabolism

Organic pollutants are carbonized chemical substances that can cause environmental pollution from a wide range of sources, including agricultural activities (such as pesticides and fertilizers), industrial production (such as solvents, plasticizers, flame retardants, and other chemicals), domestic wastewater (such as detergents and personal care products), and the burning of oil and coal. Organic pollutants can be divided into various types according to their chemical properties and sources, containing BPs, PAHs, PCBs, organochlorine pesticides (such as dichlorodiphenyltrichloroethane, DDT), organophosphorus flame retardants (OPFRs), and volatile organic compounds (VOCs) [78].

A study revealed that after two-month-old C57BL/6J male mice were exposed orally for 8 weeks to di(2-ethylhexyl) phthalate (DEHP) alone (0, 5, and 50 μg/kg/d), metabolic analyses highlighted differences in hypothalamic metabolites of males exposed to DEHP alone or in a phthalate mixture compared to control mice. These differences included lower Trp and higher NAD+ levels, indicating that DEHP may influence the metabolism of Trp in hypothalamus of mice [79]. A study on OPFRs demonstrated that 6–8 weeks old C57BL/6 prenatal mice exposure to 1 mg/kg and 5 mg/kg TPHP via oral administration for 56 days interfered with the expression of Trp-metabolizing enzyme genes, elevated 5-HT levels in the placental and fetal brain tissues of mice, disrupted the development of hippocampal 5-HTergic neurons, and ultimately induced anxiety- and depression-like behaviors in offspring [80]. Also, further tests conducted on 8-week-old C57BL/6J mice exposed to BPA at doses of 0.5, 5, and 50 mg/kg via oral administration for 22 weeks showed that BPA interfered with the expression of 5-HTM-related metabolic enzymes in both brain and colon of mice, and reduced the levels of 5-HT and 5-HIA, suggesting that BPA may affect the mutual regulation of brain–gut axis in mice by interfering with Trp metabolism [81]. A study has indicated that the exposure of C57BL/6J male adult mice to phthalates reduces the level of Trp in the brain, and the dysregulation of the KYN metabolic pathway leads to the increase in NAD+ level, the decrease in dendritic spine density and glutamate receptor protein level, and the impairment of sexual behavior [79]. In an in vitro experiment, uterine leiomyoma cells exposed to mono-(2-ethyl-5-hydroxyhexyl) phthalate (MEHHP) at doses of 0.16, 1.6, and 16 μM for 22 weeks found that phthalate significantly increased the levels of Trp and KYN and induced the expression of Trp transporters SLC7A5 and SLC7A8 as well as TDO2, thereby inhibiting the apoptosis of uterine leiomyoma cells [82]. A study on A/J mice (5- to 6-week old, female) about tobacco smoke showed that nicotine-derived nitrogenyl ketone (NNK) up-regulated IDO1 by activating transcription factor c-Jun, promoting the proliferation of regulatory T cells and inhibiting CD8+ T cells, respectively, thereby inhibiting the immune response to induce immune escape of lung epithelial tumor cells [83].

### 5.3. Effects of Microplastics and Nanoplastics on Trp Metabolism

Microplastics are defined as plastic particles with a diameter of less than 5 mm, while nanoplastics refer to even smaller fragments, typically measuring less than 1 μm. These materials originate from a wide range of sources, including microplastic particles deliberately produced during industrial processes, such as microbeads in cosmetics and microplastics in detergents, as well as tiny fragments resulting from the breakdown of larger plastic items in natural environments. Their presence is not limited to marine and freshwater systems but has also been detected in soil, air, and even polar glaciers. The potential environmental and biological impacts of microplastics and nanoplastics have garnered extensive attention. These particles can be ingested by aquatic organisms, subsequently transferring through the food chain and potentially affecting human health. Moreover, the surface of microplastics may adsorb harmful chemical substances, such as heavy metals and organic pollutants, further exacerbating their potential risks to biological health.

Several studies have shown that the exposure of adult specimens of *D. rerio* and *P. fluviatilis* to polypropylene microplastics (PP-MPs, 8–10 μm in size) at doses of 1 and 10 mg/g via water exposure for 21 days interferes with Trp metabolism in the liver, resulting in reduced metabolite levels, lipid peroxidation, DNA damage, and cell apoptosis, thereby impairing the normal function of liver cells [84]. A study has shown that when humans are exposed to excessive amounts of nano-plastics, nano-plastics enhance the hydrophobicity around the aromatic residues of tryptophan to effectively bind and change the conformation of human hemoglobin (Hb) [85]. It was found that, after exposing zebrafish embryos to 44 nm polystyrene nanoplastics (PS–NPs) at doses of 1, 10, 100 μg/L via water for 30 days, intestinal inflammation was activated, the levels of 5-HIAA, 5-HT, and 5-HTP were reduced, and the growth and development of juvenile fish were limited [86]. Through a molecular docking simulation, it was found that there is a strong affinity between polystyrene nanoplastics and Trp [87]. Additionally, mixed exposure to 2 mg/L of microplastics and heavy metals via water for 30 days can cause oxidative stress in zebrafish gills, damage the pathway of 5-HT production, and promote cell apoptosis [88]. Given the ubiquity of microplastics in food and water, future policies should prioritize reducing plastic waste, improving detection methods, and establishing safe exposure thresholds to safeguard public health. Species, dose, routes, and length of exposure of references in Section 4 are shown in Table 2.

## 6. Conclusions and Perspectives

This review reveals that environmental pollutants—including inorganic compounds, organic chemicals, and microplastics—disrupt Trp metabolism through oxidative stress, gut microbiota imbalance, and inflammatory activation, contributing to neurological disorders, immune dysregulation, and intestinal dysfunction. Here are some suggestions for future toxicological research on Trp and pollutants:

(1) Currently, the evidence regarding the impact of environmental pollutants on human health through interference with the tryptophan metabolism pathway primarily stems from in vitro studies and whole-animal experiments. Research based on population-level data remains limited, especially regarding how tryptophan metabolism mediates diseases caused by environmental pollutants. This gap is more pronounced in diverse populations with varying physiological conditions. Further investigation is warranted in this area.
(2) Despite growing recognition of these connections, critical knowledge gaps persist regarding how specific environmental pollutants alter tryptophan (Trp) metabolic pathways and subsequently affect human health. Further investigations—using in silico, in vitro, or in vivo approaches—are needed.
(3) To better investigate the relationship between Trp metabolism and environmental pollutants, integrating multi-omics data and epigenetic changes is worth considering. These approaches enable the identification of novel regulatory nodes within the Trp metabolic network that are perturbed by environmental pollutants, and serve as biomarkers or therapeutic targets for pollutant-induced disease. Advancements in in vitro models, multi-omics approaches, and computational toxicology will facilitate high-throughput screening and mechanistic studies, improving the accuracy of Trp metabolism.
(4) Strengthening environmental regulations, improving pollutant management, and promoting Trp-rich dietary interventions could mitigate health risks and support the development of targeted prevention strategies, bridging mechanistic research with practical public health policies.

## Figures and Tables

**Table 1:** Results of literature search.

Total Number of Items Retrieved	Number of Items After Removal of Duplicates and Selected on the Basis of Title and Abstract	Number of Studies Selected After Full Text Revision
200	100	88

**Table 2:** Species, dose, routes, and length of exposure of references in Section 4.

Types	References	Species and Cell Line	Dose of Exposure	Routes of Exposure	Length of Exposure
Inorganic pollutants	[69]	Human	3.08 ± 1.40 μg/L of Cd	Oral administration	15 years
	[71]	Wistar rats	0.05, 0.25, 1.25, 6.25 mg/L of As	Oral administration	30 days
	[74]	Zebrafish	0, 4, 40, 400 nM of HgCl_2_	Water exposure	96 h
	[75]	Male Fischer-344 rats	0, 0.8 ppm of ozone	Respiratory infection	4 h
	[76]	C57BL/6 male mice	25 mg/L of Pb, 10 mg/L of Hg, and 15 of mg/L Cd	Oral administration	28 days
	[77]	MIN6-K8 mouse islet β cells	0.1–1 μM of As	Cytotoxicity	30 days
Organic pollutants	[79]	C57BL/6J male mice	0, 5, 50 μg/kg/d of DEHP	Oral administration	8 weeks
	[80]	C57BL/6 prenatal mice	1, 5 mg/kg of TPHP	Oral administration	56 days
	[81]	C57BL/6J mice	0.5, 5, 50 mg/kg of BPA	Oral administration	22 weeks
	[82]	Uterine leiomyoma cell	0.16, 1.6, 16 μM of MEHHP	Cytotoxicity	48 and 72 h
	[83]	A/J female mice	50 mg/kg of NNK	Oral administration	5 weeks
Microplastics and nanoplastics	[84]	adult specimens of *D. rerio* and *P. fluviatilis*	1, 10 mg/g of 8–10 μm PP-MPs	Water exposure	21 days
	[86]	Zebrafish	1, 10, 100 μg/L of 44 nm PP-NPs	Water exposure	30 days
	[88]	Zebrafish	2 mg/L of microplastics	Water exposure	30 days

## Data Availability

No new data were created or analyzed in this study.
